# i6mA-Vote: Cross-Species Identification of DNA N6-Methyladenine Sites in Plant Genomes Based on Ensemble Learning With Voting

**DOI:** 10.3389/fpls.2022.845835

**Published:** 2022-02-14

**Authors:** Zhixia Teng, Zhengnan Zhao, Yanjuan Li, Zhen Tian, Maozu Guo, Qianzi Lu, Guohua Wang

**Affiliations:** ^1^College of Information and Computer Engineering, Northeast Forestry University, Harbin, China; ^2^College of Electrical and Information Engineering, Quzhou University, Quzhou, China; ^3^College of Information Engineering, Zhengzhou University, Zhengzhou, China; ^4^College of Electrical and Information Engineering, Beijing University of Civil Engineering and Architecture, Beijing, China; ^5^College of Bioinformatics Science and Technology, Harbin Medical University, Harbin, China

**Keywords:** N6-methyladenine, plant genomes, cross-species, feature encoding, ensemble learning

## Abstract

DNA N6-Methyladenine (6mA) is a common epigenetic modification, which plays some significant roles in the growth and development of plants. It is crucial to identify 6mA sites for elucidating the functions of 6mA. In this article, a novel model named i6mA-vote is developed to predict 6mA sites of plants. Firstly, DNA sequences were coded into six feature vectors with diverse strategies based on density, physicochemical properties, and position of nucleotides, respectively. To find the best coding strategy, the feature vectors were compared on several machine learning classifiers. The results suggested that the position of nucleotides has a significant positive effect on 6mA sites identification. Thus, the dinucleotide one-hot strategy which can describe position characteristics of nucleotides well was employed to extract DNA features in our method. Secondly, DNA sequences of Rosaceae were divided into a training dataset and a test dataset randomly. Finally, i6mA-vote was constructed by combining five different base-classifiers under a majority voting strategy and trained on the Rosaceae training dataset. The i6mA-vote was evaluated on the task of predicting 6mA sites from the genome of the Rosaceae, Rice, and Arabidopsis separately. In Rosaceae, the performances of i6mA-vote were 0.955 on accuracy (ACC), 0.909 on Matthew correlation coefficients (MCC), 0.955 on sensitivity (SN), and 0.954 on specificity (SP). Those indicators, in the order of ACC, MCC, SN, SP, were 0.882, 0.774, 0.961, and 0.803 on Rice while they were 0.798, 0.617, 0.666, and 0.929 on Arabidopsis. According to the indicators, our method was effectiveness and better than other concerned methods. The results also illustrated that i6mA-vote does not only well in 6mA sites prediction of intraspecies but also interspecies plants. Moreover, it can be seen that the specificity is distinctly lower than the sensitivity in Rice while it is just the opposite in Arabidopsis. It may be resulted from sequence similarity among Rosaceae, Rice and Arabidopsis.

## Introduction

DNA N6-methyladenine (6mA) is a methyl modification at the sixth position of the adenine ring, which was discovered by Vanyushin et al. (1968). 6mA is widely found in prokaryotes and eukaryotes (Fu et al., 2015; Greer et al., 2015; Zhang et al., 2015). It is reported that 6mA plays vital roles in DNA replication, repairing nucleotide dislocations, and preventing the invasion of foreign DNA (Wion and Casadesús, 2006). Although 6mA in animal genomes studies have been well studied, those of plants genomes have still known a little, which hampered to explore their functions. To better understand the molecular mechanism of 6mA in plants, it is the first step to determine the 6mA sites accurately.

To detect 6mA sites, several biochemical methods were developed, such as single-molecule real-time sequencing technology (SMRT-seq) (Davis et al., 2013) and restriction endonuclease-based 6mA sequencing (6MA-RE-seq) (Fu et al., 2015). In SMRT-seq, single-nucleotide molecules labeled by different fluorophores were paired with bases of a DNA sequence, and the fluorescence signals were recorded during the process of pairing. The fragment of DNA sequence may be methylated if it showed the continuous same signal during the process of pairing. 6mA-RE-seq explored restriction enzymes to fragment genomic DNA at “CATG” and “GATC” motifs that did not contain 6mA and then retained these motifs containing 6mA. In this way, after end-repair and other operations, the methylated motifs would be enriched in the internal positions of DNA fragments. However, these methods are hard to detect 6mA sites from high-throughput sequences because they are time-consuming and expensive.

Therefore, some machine learning models have been developed to identify 6mA sites in recent years because they are efficient and cheap. At first, iDNA6mA-PseKNC (Feng et al., 2019) was proposed to detect 6mA sites in the mouse genome. In this model, DNA sequences were represented by pseudo-k-tuple nucleotide composition incorporating the physicochemical properties of nucleotides, and then the sequences were classified by a support vector machine (SVM). Subsequently, i6mA-Pred (Chen et al., 2019) trained a novel SVM model to identify 6mA sites in the rice genome based on the chemical properties of nucleotide such as the loop structure, the hydrogen bond, and the amino groups, and the nucleotide frequency of DNA sequences. To avoid overfitting, i6mA-Pred used the maximum correlation maximum distance approach to select the most representative features. Afterward, iN6-methylate (Le, 2019), another novel SVM model, used FastText to generate feature vectors for DNA sequences based on the assumption that a DNA sequence is a sentence and a nucleotide is a word. Unlike previous models, MM-6mAPred (Pian et al., 2019) constructed Markov chains based on DNA sequences with 6mA sites (positive samples) and DNA sequences without 6mA sites (negative samples) in the training dataset. Based on the Markov chains, the positive and negative probabilities of a DNA sequence were calculated separately. It is considered that a sequence contained 6mA site if the ratio of positive probability against negative probability is greater than 1.

To improve the performance of above methods, ensemble learning has been increasingly applied to 6mA sites prediction. In the beginning, iDNA6mA-Rice (Lv et al., 2019), a rice 6mA site classification model based on random forest, encoded DNA sequences via three feature descriptors, namely the k-nucleotide frequency, the mono-nucleotide binary coding, and the natural vector containing the frequency, average position, and second-order central moment of mono-nucleotides. Soon afterward, on the basis of bagging with CART, i6mA-DNCP (Kong and Zhang, 2019) represented rice DNA sequences by two novel feature descriptors: dinucleotide frequency and dinucleotide physicochemical properties. In addition, i6mA-DNCP employed heuristic ideas to select the most representative features. Several months later, i6mA-Fuse (Hasan et al., 2020) was proposed to classify Rosaceae DNA sequences with random forest and linear regression. Subsequently, a random forest-based multi-species 6mA site prediction model 6mA-Finder (Xu et al., 2020) was developed, which contained three modules for mouse, rice, and a general species admixed by mouse and rice DNA sequences, respectively. i6mA-stack (Khanal et al., 2021) developed a two-level stacked ensemble classifier based on linear regression, random forest, support vector machine, and gaussian naive bayes to recognize Rosaceae 6mA sites.

With the development of deep learning, some neural network models were also developed for identifying 6mA sites. For example, iDNA6mA (Tahir et al., 2019) is composed of four layers: two convolution layers which extract features of DNA sequences, a dropout layer which is used to avoid overfitting, and a full-connection layer which performs classification tasks. Subsequently, SNNRice6mA (Yu and Dai, 2019) was improved iDNA6mA by adding a normalization layer and a pooling layer between the convolution layer and the dropout layer, which aimed to reduce redundant features of DNA sequences according to the correlation of the features. i6mA-DNC (Park et al., 2020) is similar with the above two models except it extracted features from nucleotide pairs of DNA sequences rather than from single nucleotides. It is worth noting that the three neural network models mentioned above were all developed for predicting 6mA sites in the rice genome.

Because the previously mentioned models are species-specific, Meta-i6mA (Hasan et al., 2021) was proposed for 6mA site prediction from multiple plants. Although Meta-i6mA has achieved encouraging results in intraspecies, it still has room for improvement in interspecific. To solve this problem, a novel classification model i6mA-vote was developed based on an ensemble learning strategy. In this model, DNA sequences were encoded by nucleotide position-based feature descriptors, and then these sequences were classified by an ensemble classifier integrating random forest, linear discriminant analysis, multi-layer perceptron, stochastic gradient descent, and extreme gradient boosting. The details of i6mA-vote will be introduced in the following sections.

## Materials and Methods

### Framework of i6mA-Vote

**FIGURE 1:**
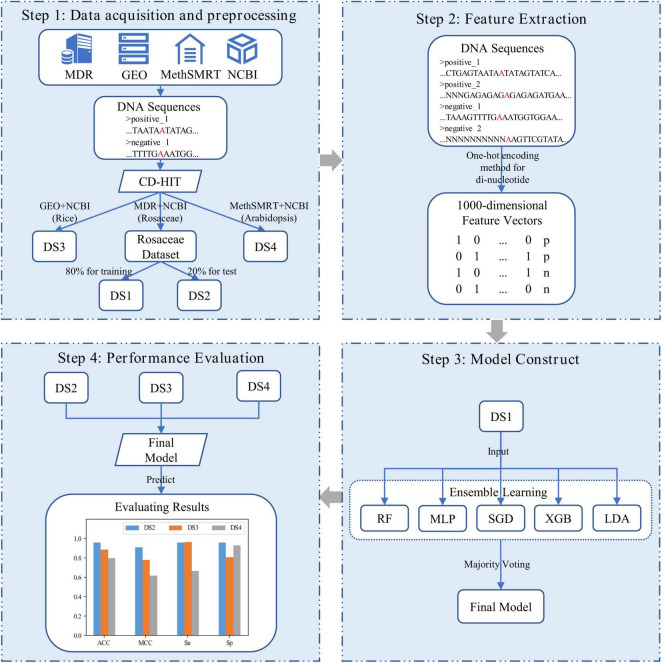
Frame diagram of i6mA-vote. (DS1, DS2, DS3, and DS4 refer to Rosaceae training dataset, Rosaceae test dataset, Rice test dataset, and Arabidopsis test dataset; In the DNA sequences, the letter “A” marked in red refers to the possible 6mA site, and the letter “N” indicates the unidentified nucleotide; In the feature vectors, “p” and “n” are short for the positive sample and the negative sample).

In our study, as shown in Figure 1, i6mA-vote was constructed by four steps. Firstly, positive samples of Rosaceae, Rice, and Arabidopsis were derived from MDR (Liu et al., 2019), GEO (Edgar et al., 2002), and MethSMRT (Ye et al., 2017) databases, and negative samples of these plants were downloaded from NCBI. For each plant, the positive and negative samples were filtered by CD-HIT (Li and Godzik, 2006) to reduce high similar samples. Then all samples were divided into three datasets according to organisms for the subsequent experiments. The Rosaceae dataset was split into a training dataset and a test dataset, and datasets for the remaining two species were used as cross-species evaluation datasets. Secondly, to transform DNA sequences into feature vectors, one-hot encoding method was performed on dinucleotides (e.g., AA, AG, …) of DNA sequences. Because the known nucleotides can be represented by four symbols (A, G, C, T) and other unknown nucleotides can be denoted by symbol N, in this way, there were twenty-five dinucleotide combinations. Thirdly, an ensemble learning model, named i6mA-vote, was built by integrating random forest (RF), multi-layer perceptron (MLP), stochastic gradient descent (SGD), linear discriminant analysis (LDA), extreme gradient boosting (XGB), based on majority voting strategy. Then all samples were represented by feature vectors and the ensemble learning model was trained on the samples. Finally, to evaluate the performance of the model, i6mA-vote was used to perform simulation a task on test datasets, and its superiority was demonstrated by accuracy, Matthew correlation coefficient, sensitivity, and specificity. In the following sections, the detail process of constructing the i6mA-vote model will be illustrated step by step.

### Datasets

The quality of the dataset affects the performance of the classification model. In this study, four high-quality datasets that have been applied in the 6mA prediction domain were selected.

The Rosaceae dataset was collected, collated, and constructed by Hasan’s team (Hasan et al., 2021). The part containing 6mA were derived from the MDR database (Liu et al., 2019). After removing similar sequences and excluding 90% sequence identity, 36,537 positive samples were obtained. The other part, including the same number of negative ones, was taken from NCBI, and it was generated by chromosomes with no 6mA detected. Finally, 80% of this dataset was randomly selected as the training dataset (DS1), and the remaining 20% was regarded as the test dataset (DS2).

The Rice dataset (DS3) was created by Lin’s group (Lv et al., 2019). The positive portion and the negative one were obtained from the GEO database (Edgar et al., 2002) and NCBI. And they both included 154000 samples.

The Arabidopsis dataset (DS4) was also constructed by Hasan’s team (Hasan et al., 2021). It extracted 31,873 6mA sites from the MethSMRT database (Ye et al., 2017) and replenished the same number of negative samples from NCBI using the same way as for the Rosaceae dataset.

Among them, DS1 was used for training the model, DS2, DS3, and DS4 were used to evaluate the generalization performance and cross-species prediction ability of the model.

All the above four datasets were downloaded from the online server of model Meta-i6mA (Hasan et al., 2021)^1^. In addition, these datasets were also processed as follows: (1) Sequences longer than 41bp were removed. (2) If a sequence was repeated multiple times, it would be deleted, leaving only one copy. (3) If a sequence was present in both positive and negative samples, it would be removed from both parts. Finally, the number of samples included in each dataset is shown in Table 1. Their sequences all consisted of 41 nucleotides with an “A” in the middle.

**TABLE 1:** Number of samples in each dataset.

Datasets	Number of positive samples	Number of negative samples	Total number
DS1	29237	29433	58670
DS2	7298	7300	14598
DS3	153635	153629	307264
DS4	31414	31843	63257

### Feature Extraction

To convert DNA sequences into feature vectors, One-hot encoding method for dinucleotides was employed in our model. This strategy and other concerned strategies will be described in detail below.

#### Our Encoding Strategy

One-hot encoding method for dinucleotides (One-hot2) is based on the one-hot encoding method in natural language processing. The one-hot encoding method compiles a dictionary using the words in the sentences and then encodes each word into a 0-1 vector through this dictionary. The length of the vector is equal to that of the dictionary, and each bit in the vector corresponds to a word in the dictionary. When encoding a word, its corresponding bit is set to 1 in the vector, and the other bits are kept at 0. Similarly, One-hot2 treats DNA sequences as sentences and dinucleotides as words.

A DNA sequence is usually composed of four standard nucleotide symbols: A, C, G, and T. However, sometimes the DNA sequence also include non-standard nucleotide symbol N, which means that the nucleotide was not identified. Accordingly, a DNA sequence may consist of 5 symbols, and it contains 25 possible symbol combinations of dinucleotides like AA, AC, AN. In our method, the one-hot2 encoded each dinucleotide into a 25-dimensional 0-1 vector. The vector of each dinucleotide is shown in Formula (1).


(1) $$\left\{ \begin{array}{c} A\,A=\left(1,0,0,0,0,0,0,0,0,0,0,0,0,0,0,0,0,0,0,0,0,0,0,0,0\right)\\ A\,C=\left(0,1,0,0,0,0,0,0,0,0,0,0,0,0,0,0,0,0,0,0,0,0,0,0,0\right)\\ A\,G=\left(0,0,1,0,0,0,0,0,0,0,0,0,0,0,0,0,0,0,0,0,0,0,0,0,0\right)\\ \vdots \\ N\,T=\left(0,0,0,0,0,0,0,0,0,0,0,0,0,0,0,0,0,0,0,0,0,0,0,1,0\right)\\ N\,N=\left(0,0,0,0,0,0,0,0,0,0,0,0,0,0,0,0,0,0,0,0,0,0,0,0,1\right) \end{array}\right.$$

To show how One-hot2 encodes DNA sequences, an example is given below. DNA sequence *D* = *ACGTNA* can be split into five dinucleotides (AC, CG, GT, TN, NA), and then they are replaced with their corresponding one-hot codes. In this way, a vector with the dimension of 125 is generated.

Because the length of the DNA sequences in our datasets are 41bp, the sequences can be spliced into 40 dinucleotides and thus the vectors of these dinucleotides were concatenated into a 1000-dimensional feature vector to describe their primary sequence.

There are three reasons why One-hot2 was chosen: (1) It can solve the problem that classifiers are not good at handling continuous data. In addition, it generates sparse vectors, allowing many machine learning problems to be linearly separated and models more efficient to be stored. (2) It considers the relationship between adjacent nucleotides as it is encoded in dinucleotide. (3) Some studies (Chen et al., 2019; Feng et al., 2019) found position-specific features can better represent sequences containing 6mA sites, and One-hot2 happens to be this kind of method.

#### The Concerned Encoding Strategies

##### Density-Based Approach

Accumulated Mono-Nucleotide Frequency (AMNF) represent the frequency of single nucleotides in the subsequence which ranges from the first nucleotide to the current nucleotide of the original sequence. Similarly, Accumulated Di-Nucleotide Frequency (ADNF) (Chen et al., 2017) denotes the nucleotide pairs which appears before current nucleotide. For example, DNA sequence *D* = *ACGTNA* can be encoded as (1, 0.5, 0.33, 0.25, 0.2, 0.33) and (1, 0.5, 0.33, 0.25, 0.2) by AMNF and ADNF, respectively.

##### Physicochemical-Properties-Based Approach

Dinucleotide Physical-Chemical Properties (DPCP) and Trinucleotide Physical-Chemical Properties (TPCP) (Manavalan et al., 2019; Wei et al., 2019) replace the DNA sequences with the vectors calculated by Equation (2) using the physicochemical-properties in Supplementary Tables 1,2. In Supplementary Table 1, the columns represent 15 physicochemical properties, and the rows represent 25 dinucleotides. In Supplementary Table 2, the columns represent 11 physicochemical properties, and the rows represent 125 trinucleotides.


(2) $$x\,P\,C\,P_{i}=N_{i}\times x\,P\,C_{i\,j}$$

where *x* = *D* refers to Dinucleotide and *x* = *T* denotes Trinucleotide. When *x* = *D*, the values of *i* range from 1 to 25, the values of *j* range from 1 to 15, *DPCP*_*i*_is the DPCP value of the *i*th dinucleotides, *N_i* is the count of the *i*th dinucleotides in the DNA sequence, and *DPC*_*ij*_ is the *j*th properties of the *i*th dinucleotides; When *x* = *T*, the values of *i* range from 1 to 125, the values of *j* range from 1 to 11, *TPCP*_*i*_ is the TPCP value of the *i*th trinucleotides, *N_i* is the count of the *i*th trinucleotides in the DNA sequence, and *TPC*_*ij*_ is the *j*th properties of the *i*th trinucleotides.

##### Position-Based Approach

One-hot encoding method for mononucleotide (One-hot1) is similar to One-hot2, except that its encoding unit is the mononucleotide. It converts a mononucleotide into a one-hot code with a length of five, corresponding to five mononucleotides (A, C, G, T, and N). For instance, the encoded vector of DNA sequence*D* = *ACGTNA* is (1,0,0,0,0| 0,1,0,0,0| 0,0,1,0,0| 0,0,0,1,0| 0,0,0,0,1| 1,0,0,0,0).

### Classifier

To train a classification model with stable and good performance, five machine learning algorithms was utilized to construct five base-classifiers. Subsequently, majority voting was adopted to integrate these five base-classifiers. Its detailed procedure is illustrated in the following steps.

(1) The processed training dataset was inputted into five machine learning algorithms, and five base-classifiers were generated. These five algorithms were random forest (RF), multi-layer perceptron (MLP), stochastic gradient descent (SGD), linear discriminant analysis (LDA), extreme gradient boosting (XGB). Among them, RF refers to one type of classifier that utilizes multiple decision trees to train and predict samples. MLP, as a simple neural network, contains three fully connected layers, the input layer, the hidden layer, and the output layer. SGD is a kind of support vector machine model. LDA is a classifier generated according to Bayes’ rule. XGB is also based on trees, but unlike random forests, its trees are regressive, and it also optimizes the algorithm itself, the efficiency and robustness of the algorithm.

(2) The five base classifiers were combined into one ensemble classifier by majority voting. That is, when three or more base classifiers judge a sequence to be a positive (or negative) sample, then their combination also treats this sequence as a positive (or negative) sample.

It should be noted that the hyperparameters of the base-learners were optimized by grid search strategy. After manually specifying variation ranges of hyperparameters, this strategy adopted an exhaustive method-like approach to find the best-performing combination from these hyperparameters. In addition, all classifier algorithms in this paper were implemented by sklearn (Hinton, 1989; Belhumeur et al., 1997; Platt, 2000; Breiman, 2001; Bengio and Glorot, 2010; Pedregosa et al., 2011; Kingma and Ba, 2014; He et al., 2015; Chen and Guestrin, 2016).

### Performance Evaluation

Our model was validated according to accuracy (ACC), Matthew correlation coefficient (MCC), Sensitivity (SN), Specificity (SP) which had been widely adopted in the field of bioinformatics (Huang and Gong, 2020; Liu et al., 2020; Smolarczyk et al., 2020; Wang H. et al., 2020; Wang J. et al., 2020; Shao and Liu, 2021; Zhang et al., 2021). These metrics can be calculated by equations (3) ∼ (6).


(3) $$A\,C\,C=\frac{n_{T\,P}+n_{T\,N}}{n_{T\,P}+n_{F\,N}+n_{T\,N}+n_{F\,P}}$$

(4) $$M\,C\,C=\frac{n_{T\,P}\times n_{T\,N}-n_{F\,N}\times n_{F\,P}}{\sqrt{\left(n_{T\,P}+n_{F\,P}\right)\times \left(n_{T\,P}+n_{F\,N}\right)\times \left(n_{T\,N}+n_{F\,P}\right)\times \left(n_{T\,N}+n_{F\,N}\right)}}$$

(5) $$S\,N=\frac{n_{T\,P}}{n_{T\,P}+n_{F\,N}}$$

(6) $$S\,P=\frac{n_{T\,N}}{n_{T\,N}+n_{F\,P}}$$

Where *TP* and *TN* refer to correctly predicted 6mA and non-6mA; *FP* and *FN* denote incorrectly predicted non-6mA and 6mA; *n_x* means the number of *x*.

## Results and Discussion

### DNA Sequence Logos

To find optimal features of samples, the DNA sequences of samples should be analyzed. Since these sequences were of equal length, they could be analyzed sequence logos (Schneider and Stephens, 1990). Two Sample Logo was employed (Vacic et al., 2006), which calculated the statistical difference between positive and negative samples at specific positions. The logo consists of three parts, the upper and lower parts represent the enriched and depleted nucleotides at specific positions, and the middle part denotes the consistent results of positive and negative samples. The x-axis indicates the position. The length of DNA sequences in our datasets is 41bp, so there are 41 scales on the *x*-axis. Additionally, as the middle nucleotide is consistent in both positive and negative samples, it is set to the 0th scale. The y-axis represents the amount of information at the position. The higher the symbol in a position, the more information the position contains. In addition, the relative size of a base letter shows its relative frequency at one position. If a letter is larger than the other letters in the column, it has a high frequency in that position. At each position, the base letters are arranged in the order of dominance from top to bottom. Generally, the consensus motif can be found by reading the top of each position.

**FIGURE 2:**
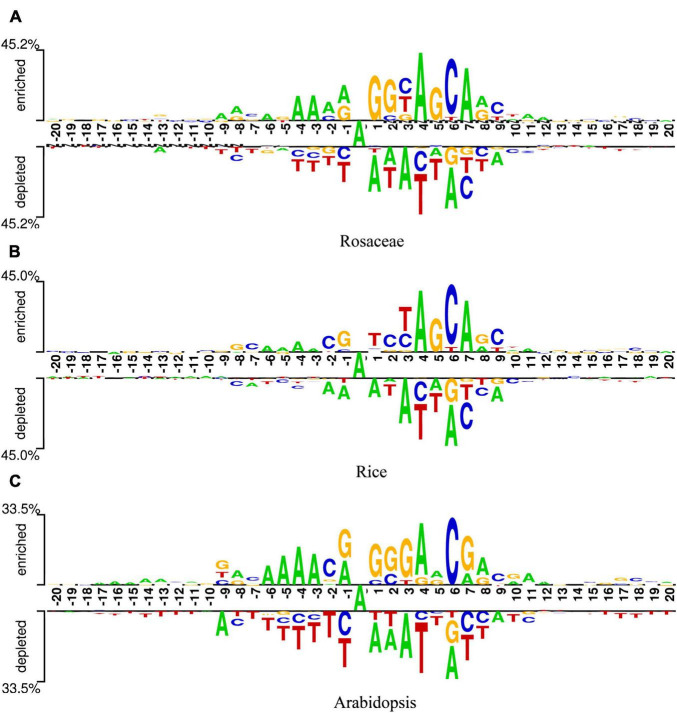
Sequence logos of Rosaceae **(A)**, Rice **(B),** and Arabidopsis **(C)**.

Figures 2A-C are the sequence logos established for Rosaceae, Rice, and Arabidopsis. From the three figures, it can be seen that the sequences have a length of 41bp with “A”s at the center. In addition, “A” enriched at positions −6, −4, −3, 4, 7, 8, 10, 11, 12, “C” enriched at positions −7, −2, 2, 6, 9, “G” enriched at positions −8, −1, 2, 3, 5, 8, and “T” enriched at positions 3. Since these sequences containing 6mA are enriched with some nucleotides at some positions, it is speculated that position-based approaches are more suitable for extracting information from the sequences in our datasets.

### Performance Evaluation of Models

To verify the conjecture in the previous section, six methods were chosen to extract the datasets as features and then they were applied to five commonly used well-performing algorithms in sklearn. Since the conjecture is too intuitive and may lead to some significant features being overlooked, not only nucleotide position-based methods are compared, but also density-based and physicochemical property-based methods were also compared.

The experimental results of 5-fold cross-validation are displayed in Table 2. The columns indicate the feature extraction methods which have been introduced in the “Feature Extraction” section. The rows denote classifier algorithms and their evaluation metrics, and they have been briefly described in the “Classifiers” section and the “Performance Evaluation” section.

**TABLE 2:** Indicators of different features and classifier algorithms.

		AMNF	ADNF	DPCP	TPCP	One-hot1	One-hot2
Random forest	ACC	0.786	0.642	0.594	0.669	0.935	**0.938**
	SN	0.746	0.627	0.587	0.683	0.937	**0.939**
	SP	0.825	0.655	0.602	0.656	0.933	**0.937**
	MCC	0.573	0.283	0.189	0.339	0.870	**0.877**
Linear discriminant analysis	ACC	0.643	0.609	0.614	0.660	0.908	**0.931**
	SN	0.650	0.624	0.597	0.629	0.937	**0.945**
	SP	0.637	0.594	0.632	0.692	0.879	**0.917**
	MCC	0.287	0.219	0.228	0.321	0.818	**0.862**
Multi-layer perceptron	ACC	0.755	0.627	0.602	0.625	0.937	**0.939**
	SN	0.743	0.602	0.576	0.621	0.936	**0.942**
	SP	0.767	0.652	0.628	0.629	**0.939**	0.937
	MCC	0.510	0.255	0.204	0.250	0.875	**0.878**
Stochastic gradient descent	ACC	0.643	0.605	0.549	0.577	0.910	**0.931**
	SN	0.631	0.647	0.561	0.481	0.917	**0.936**
	SP	0.654	0.563	0.538	0.672	0.904	**0.926**
	MCC	0.287	0.212	0.099	0.157	0.821	**0.861**
Extreme gradient boosting	ACC	0.790	0.647	0.616	0.673	**0.944**	0.940
	SN	0.788	0.650	0.617	0.672	**0.948**	0.942
	SP	0.791	0.644	0.616	0.675	**0.939**	0.937
	MCC	0.579	0.294	0.233	0.346	**0.888**	0.880

*Bold values indicate the best performance.*

As can be seen in Table 2, whichever classifier algorithm is selected, the ACCs, SNs, SPs, and MCCs of AMNF, ADNF, DPCP and TPCP are all lower than 0.80, 0.79, 0.83, and 0.60, whereas them of One-hot1 and One-hot2 are all higher than 0.93, 0.93, 0.91, and 0.86. These illustrate that compared with density-based and physicochemical property-based approaches, position-based ways can better express the characteristics contained in DNA sequences in our datasets. XGB performed slightly better with one-hot1 than one-hot2. This may be because XGB may lose some valuable information when it was applied on high-dimensional one-hot2 features. Specifically, XGB divides the high-dimensional feature space into many small parts which may be treated as noise. In addition, if the feature descriptor is One-hot1 or One-hot2, all classifiers show good performance, which indicates that all these algorithms are appropriate for this classification task.

**FIGURE 3:**
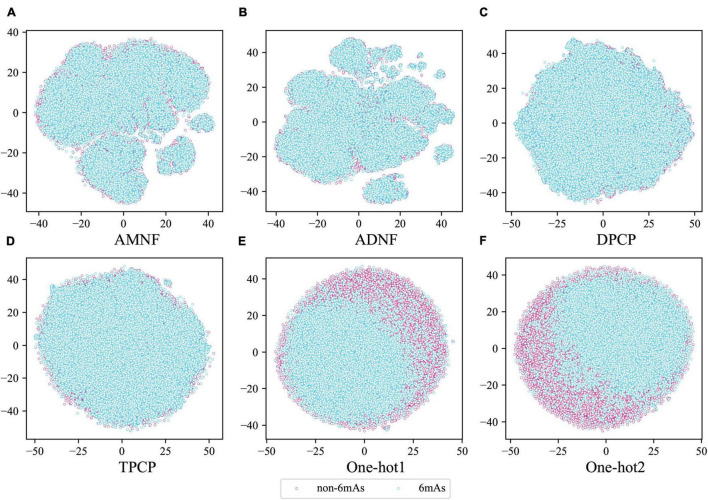
The tSNE scatterplots of AMNF **(A)**, ADNF **(B)**, DPCP **(C)**, TPCP **(D)**, One-hot1 **(E)**, and One-hot2 **(F)**. (Blue and pink dots indicate DNA sequence samples with and without 6mA sites, respectively).

Moreover, to judge intuitively whether the above six feature extraction methods were good at distinguishing between positive and negative samples, the tSNE (van der Maaten and Hinton, 2008) technique in sklearn (Pedregosa et al., 2011) was used to project the sample points of these methods from the high-dimensional space to the two-dimensional space. If the positive and negative sample points can be well separated in the two-dimensional space, they are also separable in the high-dimensional space. The visualization plots of the projection are shown in Figure 3. It can be seen from Figure 3 that the samples of the two labels are separated by certain dividing lines in Figures 3E,F, while in other subgraphs, the negative sample points are almost covered by the positive ones. These illustrate that One-hot1 and One-hot2 can better discriminate the sample points of the two labels in a high dimensional space than the other four methods.

Through these arguments, the nucleotide position-based methods are indeed more suitable for extracting features from DNA sequences in our datasets, and the assumptions that was made in the previous section are proved to be correct. Therefore, in the subsequent analysis, only One-hot1 and One-hot2 would be considered.

### Comparison of Features

**FIGURE 4:**
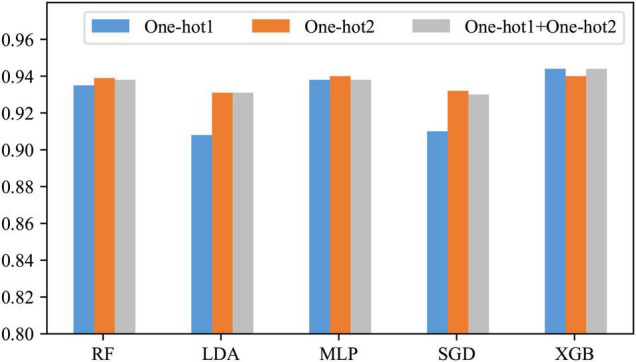
Comparison before and after feature fusion.

In the previous section, it has been learned that the position-based approaches express the information contained in our DNA sequences well. However, it is not sure which is the best among One-hot1, One-hot2, and their fusion. Therefore, in this subsection, they are compared. The comparison results are shown in Figure 4.

As can be seen from Figure 4, only when the classifier is XGB, the effect of the other two is slightly better than One-hot2; when the classifier is RF, LDA, MLP, or SGD, One-hot2 is significantly better than One-hot1 and slightly better than the fusion. The reason for this is that when encoding a dinucleotide, some information about the mononucleotide is involved. Therefore, in most cases, One-hot1 is not as good as One-hot2, and their fusion produces some redundant information. Consequently, One-hot2 is the best answer.

### Efficiency of Ensemble Strategy

**FIGURE 5:**
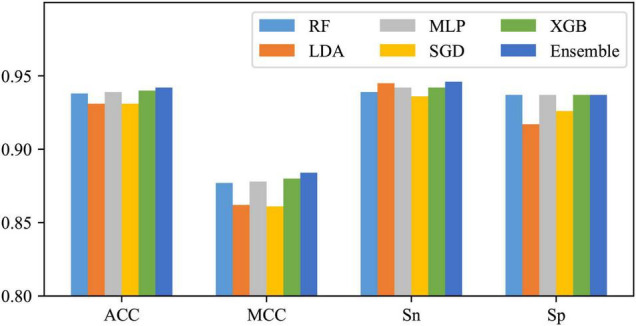
Effects of the ensemble strategy.

Using One-hot2 to extract features and take RF, LDA, MLP, SGD, and XGB as classifiers, five base models can be obtained. As shown in Figure 5, except for some differences between SN and SP of LDA and SGD, SN and SP for the other three classifiers do not differ much, as well as these base models are all with excellent performance, so they were tried to be combined with the majority voting strategy. The integrated results are also shown in Figure 5. It can be found that after voting, except for no enhancement in SP, all the other three metrics improved, which means that after this operation, the performance of the whole classification system has been risen to a higher level.

### Comparison With Other Machine Learning Models

To evaluate the generalization capability and cross-species identification ability of our model, it was applied to three test datasets, DS2, DS3, and DS4. Moreover, the test results were compared with several other machine learning models to demonstrate the advantages of our model. Table 3 shows the comparative results on Rosaceae, Rice, Arabidopsis. The columns indicate four evaluation indicators that have been introduced in the “Performance Evaluation” section. The rows represent the species and the models applied on these species. The models include Meta-i6mA (Hasan et al., 2021), i6mA-Fuse (Hasan et al., 2020), i6mA-stack (Khanal et al., 2021), i6mA-Pred (Chen et al., 2019), iDNA6mA-Rice (Lv et al., 2019), MM-6mAPred (Pian et al., 2019), and 6mA-Finder (Xu et al., 2020). Among them, i6mA-Fuse consists of two modules, which were trained by the datasets of Fragaria Vesca and Rosa Chinensis, respectively. To better distinguish them, i6mA-Fuse_FV and i6mA-Fuse_RC are used instead. The same situation is true for i6mA-stack.

**TABLE 3:** Comparison with other machine learning models on Rosaceae, Rice, and Arabidopsis.

		ACC	MCC	SN	SP
Rosaceae	Meta-i6mA	0.953	0.905	0.954	0.951
	i6mA-Fuse_FV	0.943	0.887	0.924	**0.962**
	i6mA-Fuse_RC	0.893	0.786	0.890	0.895
	i6mA-stack_FV	0.928	0.856	0.928	0.927
	i6mA-stack_RC	0.899	0.798	0.920	0.877
	i6mA-Pred	0.840	0.684	0.897	0.782
	iDNA6mA-Rice	0.878	0.764	0.951	0.805
	MM-6mAPred	0.873	0.758	**0.961**	0.785
	6mA-Finder	0.846	0.701	0.928	0.764
	i6mA-vote	**0.955**	**0.909**	0.955	0.954
Rice	Meta-i6mA	0.880	0.768	0.957	0.802
	i6mA-Fuse_FV	**0.890**	**0.781**	0.921	**0.859**
	i6mA-Fuse_RC	0.775	0.571	0.907	0.644
	i6mA-stack_FV	0.876	0.756	0.938	0.815
	i6mA-stack_RC	0.813	0.640	0.915	0.712
	i6mA-Pred	0.791	0.592	0.878	0.705
	iDNA6mA-Rice	0.755	0.561	0.960	0.547
	MM-6mAPred	0.834	0.689	0.958	0.710
	6mA-Finder	0.809	0.636	0.928	0.690
	i6mA-vote	0.882	0.774	**0.961**	0.803
Arabidopsis	Meta-i6mA	0.787	0.600	0.636	0.936
	i6mA-Fuse_FV	0.749	0.542	0.545	**0.949**
	i6mA-Fuse_RC	0.757	0.534	0.615	0.897
	i6mA-stack_FV	0.770	0.570	0.604	0.933
	i6mA-stack_RC	0.751	0.514	0.634	0.865
	i6mA-Pred	0.730	0.462	0.679	0.780
	iDNA6mA-Rice	0.734	0.473	0.655	0.812
	MM-6mAPred	0.765	0.531	**0.784**	0.747
	6mA-Finder	0.724	0.448	0.741	0.706
	i6mA-vote	**0.798**	**0.617**	0.666	0.929

*Bold values indicate the best performance.*

As can be seen from Table 3, when the species is Rosaceae, although our SN and SP values only rank second, our ACC and MCC values are the maximum, suggesting that our model has the best overall performance in Rosaceae. It can be concluded that our model can make cross-species predictions for Rice as all four metrics of our model rank at the top. And it can better find 6mA sites from unknown Rice sequences because our model has the highest SN value. Like Rosaceae, our model predicts 6mA sites well in Arabidopsis, and with the highest SP, our model can better screen out those sequences that do not contain 6mA sites. Considering the comparative results on the three species, our model has better generalization performance and cross-species prediction ability than other methods. This may be because only the best-performing feature descriptor was selected to represent the DNA sequences rather than the fusion of several well-performing features. Thereby, the risk of generating irrelevant and redundant features is reduced so that our model has better predictive performance. Furthermore, for Rosaceae, SN is approximately equal to SP and greater than 0.9, indicating that our model has a good discrimination between 6mAs and non-6mAs in the same plant family. For Rice, the SN is greater than 0.9, while the SP is less than 0.9, which may be due to a strong similarity between Rice sequences and Rosaceae positive sequences, resulting in a high false-positive rate and a low true-negative rate when the model recognizes Rice. The situation for Arabidopsis is contrary to that for Rice. It may be because the similarity between Arabidopsis sequences and Rosaceae positive sequences is weak, leading to some 6mAs in Arabidopsis being identified as non-6mAs.

## Conclusion

In this study, a plant cross-species 6mA site recognition model was constructed by ensemble learning. It has been applied on Rosaceae, Rice, and Arabidopsis and achieved good results. In the construction process, a hypothesis was put forward by analyzing the sequence logos of these three plants. The conjecture was that position-based approaches were more suitable for extracting information from the sequences in our datasets. Next, the hypothesis was verified by comparing different models and observing the tSNE visualization. Then, one-hot encoding for dinucleotide was chosen to represent the datasets by contrasting two nucleotide position-based feature extraction methods and their fusion. Finally, several well-performed models were integrated to form the final classifier by majority voting. To simulate a realistic prediction task, the model was trained on Rosaceae and tested on Rosaceae, Rice, and Arabidopsis. The experimental results showed that our model was adept at predicting the 6mA sites in homologous and heterologous species. In addition, it was also found that there might be a strong similarity between Rice sequences and Rosaceae positive sequences, and the similarity between Arabidopsis sequences and Rosaceae positive sequences is weak. The comparison with other models also showed the superiority of our model. In summary, i6mA-vote outperformed other concerned methods in predicting 6mA sites in the plant genomes. Meanwhile, our research also has the limitation that only three plants were considered. Therefore, future studies will focus on the 6mA site formation characteristics of more plants.

## Data Availability Statement

The datasets presented in this study can be found in online repositories. The names of the repository/repositories and accession number(s) can be found below: https://github.com/zhaozhengnan/i6mA-vote/tree/master, github.

## Author Contributions

ZXT improved the model, designed experiments and drafted the manuscript. ZZ proposed the initial idea and implemented the experiments. YL prepared all datasets for experiments. ZT analyzed experimental results. MG revised the manuscript. QL designed experiments and revised the manuscript. GW conceived the whole research process and revised the manuscript. All authors have read and approved the final manuscript.

## Conflict of Interest

The authors declare that the research was conducted in the absence of any commercial or financial relationships that could be construed as a potential conflict of interest.

## Publisher’s Note

All claims expressed in this article are solely those of the authors and do not necessarily represent those of their affiliated organizations, or those of the publisher, the editors and the reviewers. Any product that may be evaluated in this article, or claim that may be made by its manufacturer, is not guaranteed or endorsed by the publisher.

## Footnotes

1
http://kurata14.bio.kyutech.ac.jp/Meta-i6mA/download_file/Meta-6mA-datasets.zip
