# Supplementary material for: i6mA-Vote: Cross-Species Identification of DNA N6-Methyladenine Sites in Plant Genomes Based on Ensemble Learning With Voting
Source: Front Plant Sci. 2022 Feb 14;13:845835. doi: 10.3389/fpls.2022.845835 (PMC8882731; doi:10.3389/fpls.2022.845835)
Supplement: Supplementary file 1 [file Table_1.DOCX]

Supplementary Material

# Supplementary Tables

Supplementary Table S1. Physicochemical properties of dinucleotide.

|  | F-roll | F-tilt | F-twist | F-slide | F-shift | F-rise | roll | tilt | twist | slide | shift | rise | energy | enthalpy | entropy |
| --- | --- | --- | --- | --- | --- | --- | --- | --- | --- | --- | --- | --- | --- | --- | --- |
| AA | 0.04 | 0.08 | 0.07 | 6.69 | 6.24 | 21.34 | 1.05 | -1.26 | 35.02 | -0.18 | 0.01 | 3.25 | -1 | -7.6 | -21.3 |
| AC | 0.06 | 0.07 | 0.06 | 6.8 | 2.91 | 21.98 | 2.01 | 0.33 | 31.53 | -0.59 | -0.02 | 3.24 | -1.44 | -8.4 | -22.4 |
| AG | 0.04 | 0.06 | 0.05 | 3.47 | 2.8 | 17.48 | 3.6 | -1.66 | 32.29 | -0.22 | -0.02 | 3.32 | -1.28 | -7.8 | -21 |
| AT | 0.05 | 0.1 | 0.07 | 9.61 | 4.66 | 24.79 | 0.61 | 0 | 30.72 | -0.68 | 0 | 3.21 | -0.88 | -7.2 | -20.4 |
| AN | 0 | 0 | 0 | 0 | 0 | 0 | 0 | 0 | 0 | 0 | 0 | 0 | 0 | 0 | 0 |
| CA | 0.04 | 0.06 | 0.05 | 2 | 2.88 | 14.51 | 5.6 | 0.14 | 35.43 | 0.48 | 0.01 | 3.37 | -1.45 | -8.5 | -22.7 |
| CC | 0.04 | 0.06 | 0.06 | 2.99 | 2.67 | 14.25 | 4.68 | -0.77 | 33.54 | -0.17 | 0.03 | 3.36 | -1.84 | -8 | -19.9 |
| CG | 0.04 | 0.06 | 0.05 | 2.71 | 3.02 | 14.66 | 6.02 | 0 | 33.67 | 0.44 | 0 | 3.29 | -2.17 | -10.6 | -27.2 |
| CT | 0.04 | 0.06 | 0.05 | 3.47 | 2.8 | 17.48 | 3.6 | -1.66 | 32.29 | -0.22 | -0.02 | 3.32 | -1.28 | -7.8 | -21 |
| CN | 0 | 0 | 0 | 0 | 0 | 0 | 0 | 0 | 0 | 0 | 0 | 0 | 0 | 0 | 0 |
| GA | 0.05 | 0.07 | 0.06 | 4.27 | 3.58 | 18.41 | 2.44 | 1.44 | 35.67 | -0.05 | -0.01 | 3.3 | -1.3 | -8.2 | -22.2 |
| GC | 0.05 | 0.07 | 0.06 | 4.21 | 2.66 | 17.31 | 1.7 | 0 | 34.07 | -0.19 | 0 | 3.27 | -2.24 | -9.8 | -24.4 |
| GG | 0.04 | 0.06 | 0.06 | 2.99 | 2.67 | 14.25 | 4.68 | -0.77 | 33.54 | -0.17 | 0.03 | 3.36 | -1.84 | -8 | -19.9 |
| GT | 0.06 | 0.07 | 0.06 | 6.8 | 2.91 | 21.98 | 2.01 | 0.33 | 31.53 | -0.59 | -0.02 | 3.24 | -1.44 | -8.4 | -22.4 |
| GN | 0 | 0 | 0 | 0 | 0 | 0 | 0 | 0 | 0 | 0 | 0 | 0 | 0 | 0 | 0 |
| TA | 0.03 | 0.07 | 0.05 | 1.85 | 4.11 | 14.24 | 3.5 | 0 | 36.94 | 0.04 | 0 | 3.39 | -0.58 | -7.2 | -21.3 |
| TC | 0.05 | 0.07 | 0.06 | 4.27 | 3.58 | 18.41 | 2.44 | 1.44 | 35.67 | -0.05 | -0.01 | 3.3 | -1.3 | -8.2 | -22.2 |
| TG | 0.04 | 0.06 | 0.05 | 2 | 2.88 | 14.51 | 5.6 | 0.14 | 35.43 | 0.48 | 0.01 | 3.37 | -1.45 | -8.5 | -22.7 |
| TT | 0.04 | 0.08 | 0.07 | 6.69 | 6.24 | 21.34 | 1.05 | -1.26 | 35.02 | -0.18 | 0.01 | 3.25 | -1 | -7.6 | -21.3 |
| TN | 0 | 0 | 0 | 0 | 0 | 0 | 0 | 0 | 0 | 0 | 0 | 0 | 0 | 0 | 0 |
| NA | 0 | 0 | 0 | 0 | 0 | 0 | 0 | 0 | 0 | 0 | 0 | 0 | 0 | 0 | 0 |
| NC | 0 | 0 | 0 | 0 | 0 | 0 | 0 | 0 | 0 | 0 | 0 | 0 | 0 | 0 | 0 |
| NG | 0 | 0 | 0 | 0 | 0 | 0 | 0 | 0 | 0 | 0 | 0 | 0 | 0 | 0 | 0 |
| NT | 0 | 0 | 0 | 0 | 0 | 0 | 0 | 0 | 0 | 0 | 0 | 0 | 0 | 0 | 0 |
| NN | 0 | 0 | 0 | 0 | 0 | 0 | 0 | 0 | 0 | 0 | 0 | 0 | 0 | 0 | 0 |

Supplementary Table S2. Physicochemical properties of trinucleotide.

|  | bendability (DNase) | bendability (consensus) | trinucleotide GC content | nucleosome positioning | consensus (roll) | consensus (rigid) | DNase I (rigid) | molecular weight (daltons) | nucleosome (rigid) | nucleosome | DNase I |
| --- | --- | --- | --- | --- | --- | --- | --- | --- | --- | --- | --- |
| AAA | 0.1 | 0.05 | 0 | 36 | 0.06 | 0.06 | 6.88 | 7.18 | 621.4 | 0 | 7.05 |
| AAC | 1.6 | 2.65 | 1 | 6 | 2.64 | 2.64 | 5.26 | 6.27 | 621.4 | 3.7 | 4.86 |
| AAG | 4.2 | 4.7 | 1 | 6 | 4.7 | 4.7 | 4 | 4.74 | 621.4 | 5.2 | 3.99 |
| AAT | 0 | 0.35 | 0 | 30 | 0.35 | 0.35 | 6.7 | 7.24 | 621.4 | 0.7 | 6.62 |
| AAN | 0 | 0 | 0 | 0 | 0 | 0 | 0 | 0 | 0 | 0 | 0 |
| ACA | 5.8 | 5.5 | 1 | 6 | 5.49 | 5.49 | 3.52 | 3.81 | 622.4 | 5.2 | 3.99 |
| ACC | 5.2 | 5.3 | 2 | 8 | 5.32 | 5.32 | 3.62 | 4.16 | 622.4 | 5.4 | 3.88 |
| ACG | 5.2 | 5.3 | 2 | 8 | 5.31 | 5.31 | 3.63 | 4.16 | 622.4 | 5.4 | 3.88 |
| ACT | 2 | 3.9 | 1 | 11 | 3.92 | 3.92 | 4.47 | 6.03 | 622.4 | 5.8 | 3.65 |
| ACN | 0 | 0 | 0 | 0 | 0 | 0 | 0 | 0 | 0 | 0 | 0 |
| AGA | 6.5 | 4.9 | 1 | 9 | 4.89 | 4.89 | 3.88 | 3.41 | 622.4 | 3.3 | 5.09 |
| AGC | 6.3 | 6.9 | 2 | 25 | 6.88 | 6.88 | 2.68 | 3.52 | 622.4 | 7.5 | 2.69 |
| AGG | 4.7 | 5.05 | 2 | 8 | 5.05 | 5.05 | 3.78 | 4.45 | 622.4 | 5.4 | 3.88 |
| AGT | 2 | 3.9 | 1 | 11 | 3.92 | 3.92 | 4.47 | 6.03 | 622.4 | 5.8 | 3.65 |
| AGN | 0 | 0 | 0 | 0 | 0 | 0 | 0 | 0 | 0 | 0 | 0 |
| ATA | 9.7 | 6.25 | 0 | 13 | 6.27 | 6.27 | 3.05 | 1.61 | 621.4 | 2.8 | 5.38 |
| ATC | 3.6 | 4.45 | 1 | 7 | 4.44 | 4.44 | 4.15 | 5.09 | 621.4 | 5.3 | 3.94 |
| ATG | 8.7 | 7.7 | 1 | 18 | 7.72 | 7.72 | 2.19 | 2.17 | 621.4 | 6.7 | 3.14 |
| ATT | 0 | 0.35 | 0 | 30 | 0.35 | 0.35 | 6.7 | 7.24 | 621.4 | 0.7 | 6.62 |
| ATN | 0 | 0 | 0 | 0 | 0 | 0 | 0 | 0 | 0 | 0 | 0 |
| ANA | 0 | 0 | 0 | 0 | 0 | 0 | 0 | 0 | 0 | 0 | 0 |
| ANC | 0 | 0 | 0 | 0 | 0 | 0 | 0 | 0 | 0 | 0 | 0 |
| ANG | 0 | 0 | 0 | 0 | 0 | 0 | 0 | 0 | 0 | 0 | 0 |
| ANT | 0 | 0 | 0 | 0 | 0 | 0 | 0 | 0 | 0 | 0 | 0 |
| ANN | 0 | 0 | 0 | 0 | 0 | 0 | 0 | 0 | 0 | 0 | 0 |
| CAA | 6.2 | 4.75 | 1 | 9 | 4.76 | 4.76 | 3.96 | 3.58 | 621.4 | 3.3 | 5.09 |
| CAC | 6.8 | 6.65 | 2 | 17 | 6.63 | 6.63 | 2.83 | 3.24 | 621.4 | 6.5 | 3.25 |
| CAG | 9.6 | 6.9 | 2 | 2 | 6.9 | 6.9 | 2.67 | 1.67 | 621.4 | 4.2 | 4.57 |
| CAT | 8.7 | 7.7 | 1 | 18 | 7.72 | 7.72 | 2.19 | 2.17 | 621.4 | 6.7 | 3.14 |
| CAN | 0 | 0 | 0 | 0 | 0 | 0 | 0 | 0 | 0 | 0 | 0 |
| CCA | 0.7 | 3.05 | 2 | 8 | 3.06 | 3.06 | 5 | 6.81 | 622.4 | 5.4 | 3.88 |
| CCC | 5.7 | 5.85 | 3 | 13 | 5.83 | 5.83 | 3.31 | 3.87 | 622.4 | 6 | 3.54 |
| CCG | 3 | 3.85 | 3 | 2 | 3.87 | 3.87 | 4.5 | 5.44 | 622.4 | 4.7 | 4.28 |
| CCT | 4.7 | 5.05 | 2 | 8 | 5.05 | 5.05 | 3.78 | 4.45 | 622.4 | 5.4 | 3.88 |
| CCN | 0 | 0 | 0 | 0 | 0 | 0 | 0 | 0 | 0 | 0 | 0 |
| CGA | 5.8 | 7.05 | 2 | 31 | 7.07 | 7.07 | 2.57 | 3.81 | 622.4 | 8.3 | 2.25 |
| CGC | 4.3 | 5.9 | 3 | 25 | 5.89 | 5.89 | 3.28 | 4.68 | 622.4 | 7.5 | 2.69 |
| CGG | 3 | 3.85 | 3 | 2 | 3.87 | 3.87 | 4.5 | 5.44 | 622.4 | 4.7 | 4.28 |
| CGT | 5.2 | 5.3 | 2 | 8 | 5.31 | 5.31 | 3.63 | 4.16 | 622.4 | 5.4 | 3.88 |
| CGN | 0 | 0 | 0 | 0 | 0 | 0 | 0 | 0 | 0 | 0 | 0 |
| CTA | 7.8 | 5 | 1 | 18 | 5 | 5 | 3.81 | 2.67 | 621.4 | 2.2 | 5.73 |
| CTC | 6.6 | 6 | 2 | 8 | 5.98 | 5.98 | 3.22 | 3.35 | 621.4 | 5.4 | 3.88 |
| CTG | 9.6 | 6.9 | 2 | 2 | 6.9 | 6.9 | 2.67 | 1.67 | 621.4 | 4.2 | 4.57 |
| CTT | 4.2 | 4.7 | 1 | 6 | 4.7 | 4.7 | 4 | 4.74 | 621.4 | 5.2 | 3.99 |
| CTN | 0 | 0 | 0 | 0 | 0 | 0 | 0 | 0 | 0 | 0 | 0 |
| CNA | 0 | 0 | 0 | 0 | 0 | 0 | 0 | 0 | 0 | 0 | 0 |
| CNC | 0 | 0 | 0 | 0 | 0 | 0 | 0 | 0 | 0 | 0 | 0 |
| CNG | 0 | 0 | 0 | 0 | 0 | 0 | 0 | 0 | 0 | 0 | 0 |
| CNT | 0 | 0 | 0 | 0 | 0 | 0 | 0 | 0 | 0 | 0 | 0 |
| CNN | 0 | 0 | 0 | 0 | 0 | 0 | 0 | 0 | 0 | 0 | 0 |
| GAA | 5.1 | 4.05 | 1 | 12 | 4.06 | 4.06 | 4.39 | 4.21 | 621.4 | 3 | 5.26 |
| GAC | 5.6 | 5.5 | 2 | 8 | 5.52 | 5.52 | 3.5 | 3.93 | 621.4 | 5.4 | 3.88 |
| GAG | 6.6 | 6 | 2 | 8 | 5.98 | 5.98 | 3.22 | 3.35 | 621.4 | 5.4 | 3.88 |
| GAT | 3.6 | 4.45 | 1 | 7 | 4.44 | 4.44 | 4.15 | 5.09 | 621.4 | 5.3 | 3.94 |
| GAN | 0 | 0 | 0 | 0 | 0 | 0 | 0 | 0 | 0 | 0 | 0 |
| GCA | 7.5 | 6.75 | 2 | 13 | 6.76 | 6.76 | 2.75 | 2.84 | 622.4 | 6 | 3.54 |
| GCC | 8.2 | 9.1 | 3 | 45 | 9.08 | 9.08 | 1.39 | 2.45 | 622.4 | 10 | 1.31 |
| GCG | 4.3 | 5.9 | 3 | 25 | 5.89 | 5.89 | 3.28 | 4.68 | 622.4 | 7.5 | 2.69 |
| GCT | 6.3 | 6.9 | 2 | 25 | 6.88 | 6.88 | 2.68 | 3.52 | 622.4 | 7.5 | 2.69 |
| GCN | 0 | 0 | 0 | 0 | 0 | 0 | 0 | 0 | 0 | 0 | 0 |
| GGA | 6.2 | 5 | 2 | 5 | 4.99 | 4.99 | 3.82 | 3.58 | 622.4 | 3.8 | 4.8 |
| GGC | 8.2 | 9.1 | 3 | 45 | 9.08 | 9.08 | 1.39 | 2.45 | 622.4 | 10 | 1.31 |
| GGG | 5.7 | 5.85 | 3 | 13 | 5.83 | 5.83 | 3.31 | 3.87 | 622.4 | 6 | 3.54 |
| GGT | 5.2 | 5.3 | 2 | 8 | 5.32 | 5.32 | 3.62 | 4.16 | 622.4 | 5.4 | 3.88 |
| GGN | 0 | 0 | 0 | 0 | 0 | 0 | 0 | 0 | 0 | 0 | 0 |
| GTA | 6.4 | 5.05 | 1 | 6 | 5.07 | 5.07 | 3.77 | 3.47 | 621.4 | 3.7 | 4.86 |
| GTC | 5.6 | 5.5 | 2 | 8 | 5.52 | 5.52 | 3.5 | 3.93 | 621.4 | 5.4 | 3.88 |
| GTG | 6.8 | 6.65 | 2 | 17 | 6.63 | 6.63 | 2.83 | 3.24 | 621.4 | 6.5 | 3.25 |
| GTT | 1.6 | 2.65 | 1 | 6 | 2.64 | 2.64 | 5.26 | 6.27 | 621.4 | 3.7 | 4.86 |
| GTN | 0 | 0 | 0 | 0 | 0 | 0 | 0 | 0 | 0 | 0 | 0 |
| GNA | 0 | 0 | 0 | 0 | 0 | 0 | 0 | 0 | 0 | 0 | 0 |
| GNC | 0 | 0 | 0 | 0 | 0 | 0 | 0 | 0 | 0 | 0 | 0 |
| GNG | 0 | 0 | 0 | 0 | 0 | 0 | 0 | 0 | 0 | 0 | 0 |
| GNT | 0 | 0 | 0 | 0 | 0 | 0 | 0 | 0 | 0 | 0 | 0 |
| GNN | 0 | 0 | 0 | 0 | 0 | 0 | 0 | 0 | 0 | 0 | 0 |
| TAA | 7.3 | 4.65 | 0 | 20 | 4.67 | 4.67 | 4.01 | 2.96 | 621.4 | 2 | 5.85 |
| TAC | 6.4 | 5.05 | 1 | 6 | 5.07 | 5.07 | 3.77 | 3.47 | 621.4 | 3.7 | 4.86 |
| TAG | 7.8 | 5 | 1 | 18 | 5 | 5 | 3.81 | 2.67 | 621.4 | 2.2 | 5.73 |
| TAT | 9.7 | 6.25 | 0 | 13 | 6.27 | 6.27 | 3.05 | 1.61 | 621.4 | 2.8 | 5.38 |
| TAN | 0 | 0 | 0 | 0 | 0 | 0 | 0 | 0 | 0 | 0 | 0 |
| TCA | 10 | 7.7 | 1 | 8 | 7.7 | 7.7 | 2.2 | 1.45 | 622.4 | 5.4 | 3.88 |
| TCC | 6.2 | 5 | 2 | 5 | 4.99 | 4.99 | 3.82 | 3.58 | 622.4 | 3.8 | 4.8 |
| TCG | 5.8 | 7.05 | 2 | 31 | 7.07 | 7.07 | 2.57 | 3.81 | 622.4 | 8.3 | 2.25 |
| TCT | 6.5 | 4.9 | 1 | 9 | 4.89 | 4.89 | 3.88 | 3.41 | 622.4 | 3.3 | 5.09 |
| TCN | 0 | 0 | 0 | 0 | 0 | 0 | 0 | 0 | 0 | 0 | 0 |
| TGA | 10 | 7.7 | 1 | 8 | 7.7 | 7.7 | 10 | 1.45 | 622.4 | 5.4 | 3.88 |
| TGC | 7.5 | 6.75 | 2 | 13 | 6.76 | 6.76 | 2.75 | 2.84 | 622.4 | 6 | 3.54 |
| TGG | 0.7 | 3.05 | 2 | 8 | 3.06 | 3.06 | 5 | 6.81 | 622.4 | 5.4 | 3.88 |
| TGT | 5.8 | 5.5 | 1 | 6 | 5.49 | 5.49 | 3.52 | 3.81 | 622.4 | 5.2 | 3.99 |
| TGN | 0 | 0 | 0 | 0 | 0 | 0 | 0 | 0 | 0 | 0 | 0 |
| TTA | 7.3 | 4.65 | 0 | 20 | 4.67 | 4.67 | 4.01 | 2.96 | 621.4 | 2 | 5.85 |
| TTC | 5.1 | 4.05 | 1 | 12 | 4.06 | 4.06 | 4.39 | 4.21 | 621.4 | 3 | 5.26 |
| TTG | 6.2 | 4.75 | 1 | 9 | 4.76 | 4.76 | 3.96 | 3.58 | 621.4 | 3.3 | 5.09 |
| TTT | 0.1 | 0.05 | 0 | 36 | 0.06 | 0.06 | 0.1 | 7.18 | 621.4 | 0 | 7.05 |
| TTN | 0 | 0 | 0 | 0 | 0 | 0 | 0 | 0 | 0 | 0 | 0 |
| TNA | 0 | 0 | 0 | 0 | 0 | 0 | 0 | 0 | 0 | 0 | 0 |
| TNC | 0 | 0 | 0 | 0 | 0 | 0 | 0 | 0 | 0 | 0 | 0 |
| TNG | 0 | 0 | 0 | 0 | 0 | 0 | 0 | 0 | 0 | 0 | 0 |
| TNT | 0 | 0 | 0 | 0 | 0 | 0 | 0 | 0 | 0 | 0 | 0 |
| TNN | 0 | 0 | 0 | 0 | 0 | 0 | 0 | 0 | 0 | 0 | 0 |
| NAA | 0 | 0 | 0 | 0 | 0 | 0 | 0 | 0 | 0 | 0 | 0 |
| NAC | 0 | 0 | 0 | 0 | 0 | 0 | 0 | 0 | 0 | 0 | 0 |
| NAG | 0 | 0 | 0 | 0 | 0 | 0 | 0 | 0 | 0 | 0 | 0 |
| NAT | 0 | 0 | 0 | 0 | 0 | 0 | 0 | 0 | 0 | 0 | 0 |
| NAN | 0 | 0 | 0 | 0 | 0 | 0 | 0 | 0 | 0 | 0 | 0 |
| NCA | 0 | 0 | 0 | 0 | 0 | 0 | 0 | 0 | 0 | 0 | 0 |
| NCC | 0 | 0 | 0 | 0 | 0 | 0 | 0 | 0 | 0 | 0 | 0 |
| NCG | 0 | 0 | 0 | 0 | 0 | 0 | 0 | 0 | 0 | 0 | 0 |
| NCT | 0 | 0 | 0 | 0 | 0 | 0 | 0 | 0 | 0 | 0 | 0 |
| NCN | 0 | 0 | 0 | 0 | 0 | 0 | 0 | 0 | 0 | 0 | 0 |
| NGA | 0 | 0 | 0 | 0 | 0 | 0 | 0 | 0 | 0 | 0 | 0 |
| NGC | 0 | 0 | 0 | 0 | 0 | 0 | 0 | 0 | 0 | 0 | 0 |
| NGG | 0 | 0 | 0 | 0 | 0 | 0 | 0 | 0 | 0 | 0 | 0 |
| NGT | 0 | 0 | 0 | 0 | 0 | 0 | 0 | 0 | 0 | 0 | 0 |
| NGN | 0 | 0 | 0 | 0 | 0 | 0 | 0 | 0 | 0 | 0 | 0 |
| NTA | 0 | 0 | 0 | 0 | 0 | 0 | 0 | 0 | 0 | 0 | 0 |
| NTC | 0 | 0 | 0 | 0 | 0 | 0 | 0 | 0 | 0 | 0 | 0 |
| NTG | 0 | 0 | 0 | 0 | 0 | 0 | 0 | 0 | 0 | 0 | 0 |
| NTT | 0 | 0 | 0 | 0 | 0 | 0 | 0 | 0 | 0 | 0 | 0 |
| NTN | 0 | 0 | 0 | 0 | 0 | 0 | 0 | 0 | 0 | 0 | 0 |
| NNA | 0 | 0 | 0 | 0 | 0 | 0 | 0 | 0 | 0 | 0 | 0 |
| NNC | 0 | 0 | 0 | 0 | 0 | 0 | 0 | 0 | 0 | 0 | 0 |
| NNG | 0 | 0 | 0 | 0 | 0 | 0 | 0 | 0 | 0 | 0 | 0 |
| NNT | 0 | 0 | 0 | 0 | 0 | 0 | 0 | 0 | 0 | 0 | 0 |
| NNN | 0 | 0 | 0 | 0 | 0 | 0 | 0 | 0 | 0 | 0 | 0 |
